# Temporal trend of mortality due to Parkinson’s disease in older people in Brazil, 2002-2021

**DOI:** 10.1590/S2237-96222024v33e2024532.en

**Published:** 2024-12-09

**Authors:** Thamara Hubler Figueiró, Viviane Nogueira de Zorzi, Eleonora d’Orsi, Cassiano Ricardo Rech, Danúbia Hillesheim

**Affiliations:** 1 Universidade Federal de Santa Catarina, Programa de Pós-Graduação em Ciências Médicas, Florianópolis, SC, Brazil; 2 Universidade Federal de Santa Catarina, Programa de Pós-Graduação em Educação Física, Florianópolis, SC, Brazil; 3 Universidade Federal de Santa Catarina, Departamento de Saúde Pública, Florianópolis, SC, Brazil

**Keywords:** Enfermedad de Parkinson, Adulto Mayor, Estudios de Series Temporales, Mortalidad, Análisis de Regresión, Parkinson Disease, Aged, Time Series Studies, Mortality, Regression Analysis

## Abstract

**Objective:**

To describe the epidemiological profile and analyze the temporal trend of deaths due to Parkinson’s disease among the elderly in Brazil from 2002 to 2021.

**Methods:**

Descriptive and temporal trend study using data from the Mortality Information System. Annual percent change (APC) and 95% confidence intervals (95% CI) were estimated using the Prais-Winsten regression model.

**Results:**

Among the 57,723 deaths, there was a higher frequency in males (54.7%), individuals aged ≥ 80 years (57.7%), and White individuals (75.0%). Mortality trends increased in both males (APC = 3.32; 95%CI 2.49; 4.16) and females (APC = 2.81; 95%CI 1.80; 3.82); across all age groups, particularly ages 70-79 (APC = 4.93; 95%CI 2.98; 6.91); and in all Brazilian regions, especially the Northeast (APC = 6.79; 95%CI 4.35; 9.30).

**Conclusion:**

Deaths due to Parkinson’s disease were more frequent among males and the oldest age groups, with an increasing mortality trend observed over the period.

## Study contributions

**Main results:**

An increasing trend in mortality due to Parkinson’s disease was found among elderly people of both sexes, of all age groups and in all Brazilian regions in the period.

**Implications for services:**

The increase in mortality due to Parkinson’s disease implies greater demand for health care services and, consequently, contributes to the financial burden related to diagnosis and treatment of people with the disease.

**Perspectives:**

Investing in research and technologies for timely diagnosis, treatment and health recovery are crucial. Furthermore, the need exists to promote health policies that guarantee equitable access to specialized services.

## RESUMEN

**Objetivo:**

Describir el perfil epidemiológico, analizar la tendencia temporal de muertes por enfermedad de Parkinson entre ancianos, en Brasil, 2002-2021.

**Métodos:**

Estudio descriptivo y de tendencia temporal con datos del Sistema de Información de Mortalidad. La variación porcentual anual (VPA) y los intervalos de confianza del 95% (IC_95_%) se estimaron con el modelo de regresión Prais-Winsten.

**Resultados:**

De los 57.723 fallecimientos, hubo mayor frecuencia en hombres (54,7%), personas de ≥ 80 años (57,7%) de color blanco (75,0%). La mortalidad aumentó en hombres (VPA = 3,32; IC_95_% 2,49;4,16) y mujeres (VPA = 2,81; IC_95_% 1,80;3,82); en todos los grupos de edad, especialmente 70-79 años (VPA = 4,93; IC_95_% 2,98;6,91); en todas las regiones de Brasil, especialmente el Nordeste (VPA = 6,79; IC_95_%/ 4,35;9,30).

**Conclusión:**

Las muertes por EP fueron más frecuentes en hombres y en edades avanzadas, con una tendencia creciente de mortalidad durante el período.

## INTRODUCTION

Parkinson’s disease is considered the second most common degenerative disease of the central nervous system.^1^ Its main symptoms are motor symptoms, including bradykinesia, rigidity and tremor, generally beginning to manifest themselves between the ages of 65 and 70. However, rarer genetic forms can appear before the age of 40.^1^

Prevalence of Parkinson’s disease is steadily increasing, especially in low- and middle-income countries, possibly driven by an aging population and prolonged disease duration. Furthermore, Parkinson’s disease incidence rates are estimated to vary between 8 and 18 per 100,000 people per year.^3^ However, data suggest that within countries there are ethnic and regional variations in the risk of the disease, possibly influenced by inequalities in access to healthcare.^4^

A study, using World Health Organization mortality data, revealed that, between 1994 and 2019, Parkinson’s disease mortality rates from increased in both sexes globally; however, the increase was more pronounced in the male sex. Overall, the mortality rate per 100,000 inhabitants rose from 1.76 in 1994 to 5.67 in 2019, representing a 222% increase over 25 years.^5^

In Brazil, few studies have addressed mortality indicators related to Parkinson’s disease.^6,7^ A survey conducted in Brazil, from 2010 to 2019, identified 11,776 deaths in this period, with higher incidence in the male sex. However, it is important to note that the study was limited to Brazilian state capitals and included individuals aged 40 years or over.^6^ Another study analyzed Parkinson’s disease morbidity and mortality in Brazil from 2008 to 2020, considering adults and elderly people. The results revealed an average of 875 hospitalizations per year, with a higher mortality rate in the states of Rio Grande do Sul and Rio de Janeiro. These data indicate a predominance of elderly people and the male sex, with the southern region of Brazil having the highest mortality rate.^7^

Analysis of the mortality rate in elderly people over two decades, using robust statistical techniques, will make it possible to identify patterns throughout Brazil. This updated investigation is crucial for highlighting changes in the occurrence of deaths related to Parkinson’s disease in older people, and is essential for planning health services. This study will contribute to enriching existing knowledge, providing valuable information for health professionals, researchers and policymakers in the Brazilian context.

As such, the objective of this study was to describe the epidemiological profile and analyze the temporal trend of deaths due to Parkinson’s disease among elderly people in Brazil, from 2002 to 2021.

## METHODS

This is a descriptive study of the epidemiological profile of Parkinson’s disease deaths and also an ecological study analyzing the temporal trend of Parkinson’s disease mortality rates, according to sex, age group and Brazilian macro-regions, from 2002 to 2021, using data held on the Mortality Information System (*Sistema de Informação sobre Mortalidade* - SIM), made available by the Brazilian National Health System Information Technology Department (*Departamento de Informática do Sistema Único de Saúde*). 

The analysis was conducted in Brazil which, according to the Brazilian Institute of Geography and Statistics (*Instituto Brasileiro de Geografia e Estatística* - IBGE), covers an area of 8,510,417.771 km², with a total population of approximately 203,080,756 inhabitants. Of this total, 32,113,490 are elderly people aged 60 or over.

The study included death records in which Parkinson’s disease was classified as the underlying cause of death, as per code G20 of the International Statistical Classification of Diseases and Related Health Problems (ICD-10), for people aged 60 years or more. Population data and estimates for Brazil as a whole and its macro-regions were extracted from the 2010 population census, provided by IBGE. The data were extracted on August 14, 2023.

The following variables were analyzed:

- Sex (male; female);
- Age group (in years: 60-69; 70-79; 80 or over);
- Race/skin color (White; Black; Asian; mixed race; Indigenous; unknown);
- Schooling (none; 1-3 years; 4-7 years; 8-11 years; 12 years or more; unknown);
- Marital status (single; married; widowed; separated; other; unknown);
- Macro-region of residence (North; Northeast; Southeast; South; Midwest);
- Year of notification (2002 to 2021);
- Parkinson’s disease mortality rate.

Mortality rates were obtained by dividing the number of deaths from Parkinson’s disease in people aged 60 years or more by the estimated number of inhabitants in that age group, according to IBGE data for the same period. The results were multiplied by 100,000 inhabitants. Overall mortality coefficients in the elderly population were also calculated, segmented by sex, age group and Brazilian macro-regions.

The descriptive analysis of the profile of deaths due to Parkinson’s disease provided the absolute (n) and relative (%) frequencies of the variables. Parkinson’s disease mortality rates were also described. 

Time series analysis was performed using the Prais-Winsten regression model to correct the first-order autocorrelation effect, frequently found in population data.^8^ The dependent variable used in the analysis was the Parkinson’s disease mortality rate logarithm, while the independent variable was comprised of the years covered by the time series (2002-2021). The formulae proposed by Antunes and Cardoso^8^ were applied to calculate annual percentage change (APC) and 95% confidence intervals (95%CI). 

Regarding interpretation, a p-value ≥ 0.05 was interpreted as indicating time series stability, that is, absence of significant change in the trend, while a p-value 0.05 was interpreted as a significant change (positive or negative change) in the trend in time series increase or decrease.^8^

Percentage change (PC) in the mortality rate between 2002 and 2021 was calculated by applying the following formula:


$$PC=\frac{\text{Mortality rate in 2021 − Mortality rate in 2002}}{\text{Mortality rate in 2002}}\times 100$$

The data were organized using Microsoft Office Excel 2019® and then exported for analysis with the Stata 14 statistical package (StataCorp, Texas, USA). 

As public domain anonymized data were used, the study project did not need to be submitted for appraisal by a Research Ethics Committee, in accordance with National Health Council Resolution No. 510, dated April 7, 2016.

## RESULTS

In the period from 2002 to 2021, 57,723 deaths due to Parkinson’s disease were recorded for individuals aged 60 years or over in Brazil, with a higher proportion of male deaths (54.7%), in the 80 or over age group (57.7%), among elderly people of White race/skin color (75.0%), married people(41.5%) and among those with 1 to 3 years of schooling (24.4%). More than half of the deaths occurred in the Southeast region of Brazil (52.3%), followed by the Southern (20.2%) and Northeast (18.1%) regions (Table 1).

**Table 1:** Description of deaths due to Parkinson’s disease according to sociodemographic characteristics and Brazilian macro-regions, in individuals aged ≥ 60 years, Brazil, 2002-2021 (N = 57,723)

**Variables**	**N**	**%**
**Sex**		
Male	31,592	54.7
Female	26,131	45.3
**Age group (years)**		
60-69	5,519	9.6
70-79	18,891	32.7
≥ 80	33,313	57.7
**Race/skin color**		
White	43,310	75.0
Black	1,763	3.1
Asian	517	0.9
Mixed race	9,778	16.9
Indigenous	37	0.1
Unknown	2,318	4.0
**Marital status**		
Single	5,990	10.4
Married	23,944	41.5
Widowed	21,705	37.6
Separated	2,532	4.4
Other	524	0.9
Unknown	3,028	5.2
**Schooling (years)**		
None	7,399	12.8
1-3	14,092	24.4
4-7	10,725	18.6
8-11	7,454	12.9
≥ 12	5,323	9.2
Unknown	12,730	22.1
**Macro-region of residence**		
North	1,784	3.1
Northeast	10,425	18.1
Southeast	30,188	52.3
South	11,675	20.2
Midwest	3,651	6.3
Brazil	57,723	100.00

Table 2 shows the annual Parkinson’s disease mortality rates during the study period. An increase in coefficients was seen for all characteristics analyzed. Mortality coefficients in the female population increased from 6.6 per 100,000 inhabitants in 2002, to 12.0 per 100,000 inhabitants in 2021, while in the male population they increased from 10.0 per 100,000 in 2002, to 19.6 per 100,000 inhabitants in 2021, corresponding to a 96.0% increase among males (Table 2).

**Table 2:** Mortality coefficient (rates calculated per 100,000 inhabitants) due to Parkinson’s disease in individuals aged ≥60 years, by sex, age group and Brazilian marco-regions, Brazil, 2002-2021

	**2002**	**2003**	**2004**	**2005**	**2006**	**2007**	**2008**	**2009**	**2010**	**2011**	**2012**	**2013**	**2014**	**2015**	**2016**	**2017**	**2018**	**2019**	**2020**	**2021**	**2021/ 2002 (%)** ^a^
**Sex**																					
Male	10.0	10.6	11.7	13.0	14.1	14.4	14.0	13.7	14.3	15.7	16.1	15.8	16.4	15.9	16.8	17.0	17.9	19.4	19.2	19.6	+96.0
Female	6.6	7.3	7.5	9.0	10.2	9.5	9.1	10.2	10.4	9.9	10.6	10.4	11.2	10.7	10.9	10.9	12.2	12.2	12.0	12.0	+81.8
**Age group (years)**																					
60-69	1.9	1.7	2.1	2.0	2.0	2.1	2.2	2.2	2.2	2.0	2.0	2.1	2.1	2.1	2.3	2.2	2.4	2.6	2.4	2.7	+42.1
70-79	9.5	10.6	11.1	12.7	13.6	13.4	12.7	12.4	13.2	14.3	14.6	13.4	14.9	12.9	14.0	14.3	15.1	15.8	15.8	15.8	+66.3
≥ 80	34.5	37.0	37.0	43.8	49.3	47.5	44.6	47.9	48.2	49.2	52.6	53.3	55.1	55.6	56.1	56.1	60.7	63.0	62.2	61.7	+78.8
**Macro-region**																					
North	3.5	3.0	3.7	5.5	6.5	5.2	5.8	6.5	5.4	7.3	7.5	6.9	7.1	8.9	7.8	8.9	10.0	8.6	12.2	9.9	+182.9
Northeast	3.4	3.9	4.5	6.2	6.8	7.5	8.4	7.3	8.2	8.7	8.8	9.2	9.8	10.3	10.8	10.7	10.5	11.9	12.8	12.8	+276.5
Southeast	10.3	11.0	11.4	13.1	14.4	13.9	12.6	14.0	14.7	14.2	14.9	14.2	15.4	14.3	14.7	14.4	15.5	16.7	15.3	15.9	+54.4
South	11.1	12.0	12.8	13.8	15.1	14.9	14.0	14.2	14.2	15.6	16.2	16.0	16.0	14.4	16.4	16.9	19.7	18.1	18.6	19.4	+74.8
Midwest	7.8	10.0	10.7	10.4	11.9	10.6	10.7	11.7	10.3	11.5	13.1	13.5	13.0	14.3	12.9	14.4	15.8	17.6	16.5	15.1	+93.6
Brazil	8.1	8.8	9.3	10.8	11.9	11.7	11.3	11.7	12.1	12.5	13.0	12.8	13.5	13.0	13.5	13.6	14.7	15.4	15.2	15.4	+90.1

a) Percentage increase (+) or decrease (−), comparing the years 2021 and 2002.

In the 80 or over age group, mortality rates increased from 34.5 per 100,000 inhabitants in 2002, to 61.7 per 100,000 inhabitants in 2021, representing an increase of more than 78% in the period. Regarding the percentage increase in mortality rates between the end and beginning of the time series, the Northeast region stood out with a 276.5% increase in mortality rates (Table 2).

**Figure 1:**
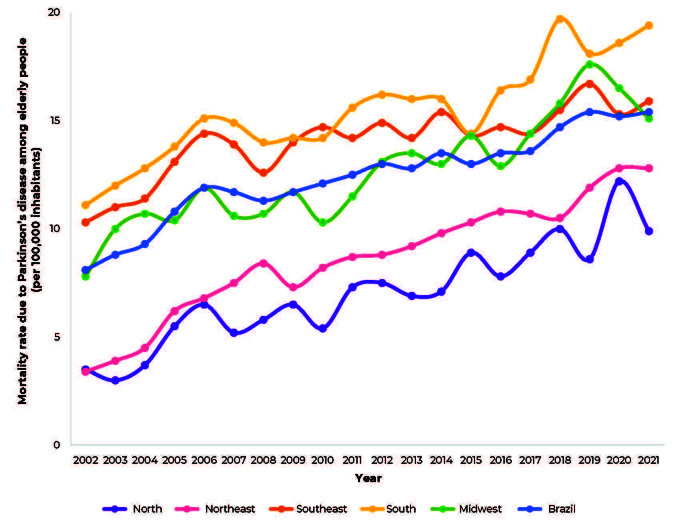
Time series of mortality coefficients due to Parkinson’s disease in individuals aged ≥ 60 years, on Brazil and Brazilian macro-regions, 2002-2021

When analyzing the Parkinson’s disease mortality coefficients in the Brazilian regions, the Southern region stood out, having the highest mortality coefficients throughout the study period (except in 2010), reaching 19.4 deaths per 100,000 inhabitants in 2021, followed by the Southeast region, where there were 10.3 deaths per 100,000 inhabitants in 2002, and 15.9 per 100,000 inhabitants in 2021 (Figure 1).

The temporal trend analysis showed an increasing trend in mortality in both sexes, in all age groups and in all Brazilian regions. The highest APC was found for males (3.32%; 95%CI 2.49;4.16), in the 70-79 age group (4.93%; 95%CI 2.98;6.91 ) and in the Northeast region (6.79%; 95%CI 4.35;9.30), followed by the Northern region, with 5.85% APC (95%CI 4.45;7.28) (Table 3).

**Table 3:** Time series of mortality coeffcients: due to Parkinson’s disease in individuals aged ≥ 60 years, by sex, age group and Brazilian macro-regions, Brazil, 2002-2021

Variable	Coefficient (logarithm)	Standard error	% (95%CI)	p-value	Interpretation
**Sex**					
Male	0.01	0.00	3.32 (2.49;4.16)	<0.001	Increase
Female	0.01	0.00	2.81 (1.80;3.82)	<0.001	Increase
**Age group (years)**					
60-69	0.00	0.00	1.49 (0.85;2.15)	<0.001	Increase
70-79	0.02	0.00	4.93 (2.98;6.91)	<0.001	Increase
≥ 80	0.01	0.00	2.93 (2.21;3.65)	<0.001	Increase
**Macro-region**					
North	0.02	0.00	5.85 (4.45;7.28)	<0.001	Increase
Northeast	0.03	0.00	6.79 (4.35;9.30)	<0.001	Increase
Southeast	0.00	0.00	1.87 (1.07;2.68)	<0.001	Increase
South	0.01	0.00	2.46 (1.77;3.16)	<0.001	Increase
Midwest	0.01	0.00	3.15 (2.44;3.87)	<0.001	Increase
Brazil	0.01	0.00	3.12 (2.18;4.07)	<0.001	Increase

## DISCUSSION

This study found a predominance of Parkinson’s disease recorded as cause of death of males, those in older age groups and with low schooling. The highest mortality rates were recorded in the Southern and Southeast regions, as well as in the 80 or over age group. The results of the temporal trend analysis indicated an increase in Parkinson’s disease mortality in both sexes, in all age groups and in all Brazilian macro-regions, with emphasis on greater annual percentage change for males, the 70-79 age group, and in the Northeast and Northern regions.

A higher proportion of deaths and higher mortality rates were found among males when compared to females. These results corroborate data from previous studies that analyzed Parkinson’s disease deaths in different periods, both nationally,^6,7^ and globally.^5^ This difference can be explained by several factors. Women naturally produce estrogens, hormones that have been shown to have a protective effect against the development of Parkinson’s disease. Additionally, men generally experience the first symptoms of the disease at a younger age than women, meaning they have a longer period for the disease to develop, resulting in an increased mortality rate.^5^

In this study, an increase of more than 78% in mortality rates due to Parkinson’s disease was found in the 80 and over age group, in the period analyzed. This finding is in line with other Brazilian studies that used the same data sources in different periods, as well as being in line with international trends.^5,6,7^ There is indeed is a trend for Parkinson’s disease prevalence to increase with age, resulting in more severe symptoms and greater risk of mortality associated with the disease.^9,10^ This phenomenon is influenced by increased life expectancy and population aging.^5,11^ Furthermore, comorbidities such as dementia, frailty and falls, common in this age group, are important predictors of mortality in elderly people with Parkinson’s disease.^12^

An inversely proportional relationship was found between Parkinson’s disease mortality and level of education, with 24.4% of deaths occurring in people with up to three years of schooling. This contrasts with other studies, which showed that high educational level and IQ are risk factors for Parkinson’s disease.^13,14^ Furthermore, our results can be partially attributed to the high number of deaths for which level of education was not reported. It should be noted that approximately 22.1% of the records did not have information on level of educational. Failure to adequately fill out this information on the SIM system may have influenced the profile we identified.

The Southern region stood out in relation to the rest of Brazil for consistently recording the highest Parkinson’s disease mortality rates in the period studied, followed by the Southeast region. These results are in agreement with data from a study that analyzed Parkinson’s disease mortality rates in a population of individuals aged 30 or over, in Brazil, from 2008 to 2020, which found higher Parkinson’s disease mortality rates in the Southern region.^7^ A study carried out only in Brazilian state capitals identified a higher mortality rate in the Southeast region during the period from 2010 to 2019, in individuals aged 40 or over.^6^

Due to the magnitude of its territory and regional diversity, Brazil has notable discrepancies in socioeconomic development, demography, access to health services (better diagnosis) and life expectancy.^15^ In particular, the Southern and Southeast regions are characterized by a high Human Development Index, which considers factors such as income and longevity.^16^ These inter-regional disparities can influence aging patterns, which are reflected in the regional differences in Parkinson’s disease mortality identified in this study. 

An increasing trend in Parkinson’s disease mortality was found in both sexes, in all age groups and in all Brazilian regions. This result can be partially explained by the improvement in the quality of records. This may influence death certificates, contributing to the continuous increase in Parkinson’s disease mortality.^17^ However, it is important to highlight other possible explanations. Globally, Parkinson’s disease has had the highest growth rate among neurological conditions, and its increasing prevalence may be having a significant impact on mortality. This increase is primarily attributed to an aging population, significant advances in diagnostic biomarkers and disease-modifying treatments, as well as environmental causes.^5^

When analyzing the APC rates for Parkinson’s disease deaths found by our study, we found that the Northeast region had the highest APC, reaching 6.79%, followed by the Northern region, with 5.85% APC. During the period from 2000 to 2014, the Northern and Northeast regions stood out for opening new agricultural frontiers and experienced the largest increases in pesticide sales, with the Northern region showing a significant increase of 99.78%, closely followed by the Northeast, which had a significant increase of 97.50%.^27^ The degree of intensity of pesticide use in the states of the Northern and Northeast regions continued to increase until 2017.^20^ Furthermore, we have already mentioned other possible explanations for higher APC in these regions, such as increased access to health services and diagnostic capacity,^15^ as well as improved information system records and population aging.

With regard to sex, there was an increase in the APC for deaths due to Parkinson’s disease in both males (3.32%) and females (2.81%). Although the disease is more prevalent in males compared to females, as evidenced both by our study and a global study, it is noteworthy that a significant increase in APC was also seen in females. Evidence demonstrates the existence of differences between the sexes in the clinical phenotype, biomarkers and therapeutic management of Parkinson’s disease.^28,29^ A literature review found that this disease in women begins with a more benign phenotype, probably due to the effect of estrogens. However, as the disease progresses, women have greater risk of developing highly disabling treatment-related complications, as well as dyskinesia, when compared to men.^29^ Additionally, women are less likely to receive effective treatment,^28^ and have greater probability of disease severity and more comorbidities than men.^30^ Together these factors may contribute to explaining the increase in APC also identified among females.

Mortality information plays a fundamental role in planning and allocating public health resources. These data are essential for supporting the development of public policies aimed at neurodegenerative diseases, enabling adaptation of health care strategies according to identified trends. In our research, an increase in Parkinson’s disease mortality rates was found in all the characteristics analyzed. Given this scenario, it is crucial to invest in strategies that enable early diagnosis, effective treatment and comprehensive support for those with the disease. 

Regarding the limitations of this study, it is important to highlight that use of secondary data can present challenges, due to possible errors and omissions in the recording of information and/or underreporting of the disease. A significant flaw in filling out the schooling level variable was identified, affecting 22.1% of the records. This may have caused classification bias, contributing to the higher percentage of deaths detected among individuals with lower education levels. Furthermore, it is important to highlight the impossibility of carrying out trend analyses for other characteristics, such as race/skin color and marital status, due to the lack of detailed population estimates that would serve as necessary denominators for calculating mortality rates. However, our research stands out for using robust methods for analyzing the temporal trend of mortality rates and for covering an extensive period of observation, from 2002 to 2021. Furthermore, the analyses were stratified by sex, age group and Brazilian macro-regions, thus enabling more detailed analysis of mortality patterns in these subgroups of the population.

In conclusion, deaths due to Parkinson’s disease predominated in the male sex, in older age groups, and in those of White race/skin color, with a trend towards mortality increasing in both sexes, all age groups and Brazilian macro-regions. As such, investments are needed in research and technologies for early diagnosis, effective treatment and health recovery. Furthermore, it is crucial to implement health policies that guarantee equitable access to specialized services, aiming to offer quality care to all health service users affected by Parkinson’s disease. 

## References

[1] Tysnes OB, Storstein A (2017). Epidemiology of Parkinson’s disease. J Neural Transm.

[2] Grotewold N, Albin RL (2024). Update: Descriptive epidemiology of Parkinson disease. Parkinsonism Relat Disord.

[3] Lee A, Gilbert RM (2016). Epidemiology of Parkinson Disease. Neurol Clin.

[4] Ben-Shlomo Y, Darweesh S, Llibre-Guerra J, Marras C, San Luciano M, Tanner C (2024). The epidemiology of Parkinson’s disease. The Lancet.

[5] Lampropoulos IC, Malli F, Sinani O, Gourgoulianis KI, Xiromerisiou G (2022). Worldwide trends in mortality related to Parkinson’s disease in the period of 1994–2019: Analysis of vital registration data from the WHO Mortality Database. Front Neurol.

[6] Viana L dos S, Fantin C (2021). Evolution of Mortality Due to Parkinson’s Disease in the Capitals of the Brazilian States in the Period from 2010 to 2019. Journal of Pharmacy and Pharmacology.

[7] Vasconcellos PRO, Rizzotto MLF, Taglietti M (2023). Morbidade hospitalar e mortalidade por Doença de Parkinson no Brasil de 2008 a 2020. Saúde em Debate.

[8] Antunes JLF, Cardoso MRA (2015). Uso da análise de séries temporais em estudos epidemiológicos. Epidemiologia e Serviços de Saúde.

[9] Collier TJ, Kanaan NM, Kordower JH (2017). Aging and Parkinson’s disease: Different sides of the same coin?. Movement Disorders.

[10] Ball N, Teo WP, Chandra S, Chapman J (2019). Parkinson’s Disease and the Environment. Front Neurol.

[11] Silva ABG, Pestana BC, Hirahata FAA, Horta FB de S, Oliveira ESBE (2021). Doença de Parkinson: revisão de literatura / Parkinson’s Disease: literature review. Brazilian Journal of Development.

[12] Macleod AD, Taylor KSM, Counsell CE (2014). Mortality in Parkinson’s disease: A systematic review and meta‐analysis. Movement Disorders.

[13] Fardell C, Torén K, Schiöler L, Nissbrandt H, Åberg M (2020). High IQ in Early Adulthood Is Associated with Parkinson’s Disease. J Parkinsons Dis.

[14] Shi J, Tian J, Fan Y (2022). Intelligence, education level, and risk of Parkinson’s disease in European populations: A Mendelian randomization study.

[15] Monteiro A, Castro CN de, Brandão CA (2017). Desenvolvimento Regional No Brasil: Políticas, Estratégias e Perspectivas.

[16] Atlas do Desenvolvimento Humano no IF, Atlas do Desenvolvimento Humano no Brasil (2024). Atlas do Desenvolvimento Humano no Brasil (AtlasBR).

[17] Shi H, Counsell C (2021). Accuracy of death certificates for recording parkinsonian syndromes and associated dementia. J Neurol.

[18] Ribeiro SD de M, Siqueira MT de, Gurgel IGD, Diniz GTN (2022). A comercialização de agrotóxicos e o modelo químico-dependente da agricultura do Brasil.

[19] Lopes CVA, Albuquerque GSC de (2018). Agrotóxicos e seus impactos na saúde humana e ambiental: uma revisão sistemática. Saúde em Debate.

[20] Moraes RF de (2019). Agrotóxicos no Brasil: padrões de uso, política da regulação e prevenção da captura regulatória.

[21] Vasconcellos PRO, Rizzotto MLF, Obregón PL, Alonzo HGA (2020). Exposição a agrotóxicos na agricultura e doença de Parkinson em usuários de um serviço público de saúde do Paraná, Brasil. Cad Saude Colet.

[22] Medeiros MS, Reddy SP, Socal MP, Schumacher-Schuh AF, Rieder CRM (2020). Occupational pesticide exposure and the risk of death in patients with Parkinson’s disease: an observational study in southern Brazil. Environmental Health.

[23] Santos A de SE, Krawczyk N, Parks CG (2021). Parkinson’s disease hospitalization rates and pesticide use in urban and non-urban regions of Brazil. Cad Saude Colet.

[24] Moura DD, Borges V, Ferraz HB (2023). History of high household pesticide use and Parkinson’s disease in Brazil. Parkinsonism Relat Disord.

[25] Ahmed H, Abushouk AI, Gabr M, Negida A, Abdel-Daim MM (2017). Parkinson’s disease and pesticides: A meta-analysis of disease connection and genetic alterations. Biomedicine Pharmacotherapy.

[26] Yan D, Zhang Y, Liu L, Shi N, Yan H (2018). Pesticide exposure and risk of Parkinson’s disease: Dose-response meta-analysis of observational studies. Regulatory Toxicology and Pharmacology.

[27] Ribeiro SD de M, Siqueira MT de, Gurgel IGD, Diniz GTN (2022). A comercialização de agrotóxicos e o modelo químico-dependente da agricultura do Brasil.

[28] Picillo M, Nicoletti A, Fetoni V, Garavaglia B, Barone P, Pellecchia MT (2017). The relevance of gender in Parkinson’s disease: a review. J Neurol.

[29] Ferreira LP de S, Silva RA da, Costa MMM da (2022). Sex differences in Parkinson’s Disease: An emerging health question. Clinics.

[30] Dahodwala N, Pei Q, Schmidt P (2016). Sex Differences in the Clinical Progression of Parkinson’s Disease. Journal of Obstetric, Gynecologic Neonatal Nursing.

