# Supplementary material for: Temporal trend of mortality due to Parkinson’s disease in older people in Brazil, 2002-2021
Source: Epidemiol Serv Saude. 2024 Dec 9;33:e2024532. doi: 10.1590/S2237-96222024v33e2024532.en (PMC11654716; doi:10.1590/S2237-96222024v33e2024532.en)
Supplement: Translation

# Translation: Tendência temporal da mortalidade por doença de Parkinson em pessoas idosas no Brasil, 2002-2021

**DOI:** 10.1590/S2237-96222024v33e2024532.pt

**Authors:** Thamara Hubler Figueiró, Viviane Nogueira de Zorzi, Eleonora d’Orsi, Cassiano Ricardo Rech, Danúbia Hillesheim

**Affiliations:** 1 Universidade Federal de Santa Catarina, Programa de Pós-Graduação em Ciências Médicas, Florianópolis, SC, Brasil; 2 Universidade Federal de Santa Catarina, Programa de Pós-Graduação em Educação Física, Florianópolis, SC, Brasil; 3 Universidade Federal de Santa Catarina, Departamento de Saúde Pública, Florianópolis, SC, Brasil

**Keywords:** Doença de Parkinson, Pessoa Idosa, Estudos de Séries Temporais, Mortalidade, Análise de Regressão

## Abstract

**Objetivo:**

Descrever o perfil epidemiológico e analisar a tendência temporal das mortes por doença de Parkinson entre pessoas idosas no Brasil, 2002-2021.

**Métodos:**

Estudo descritivo e de tendência temporal com dados do Sistema de Informação sobre Mortalidade. A variação percentual anual (VPA) e os intervalos de confiança de 95% (IC_95_%) foram estimados pelo modelo de regressão de Prais-Winsten.

**Resultados:**

Dos 57.723 óbitos identificados, observou-se maior frequência no sexo masculino (54,7%), idade ≥ 80 anos (57,7%) e raça/cor da pele branca (75,0%). Houve tendência de aumento da mortalidade nos sexos masculino (VPA = 3,32; IC_95_% 2,49;4,16) e feminino (VPA = 2,81; IC_95_%1,80;3,82); em todas as faixas etárias, com ênfase na de 70 a 79 anos (VPA = 4,93; IC_95_% 2,98;6,91); e em todas as regiões brasileiras, principalmente no Nordeste (VPA = 6,79; IC_95_% 4,35;9,30).

**Conclusão:**

Na população de estudo, os óbitos por DP foram mais frequentes no sexo masculino e nas idades mais avançadas. Houve tendência de aumento da mortalidade pela doença no período.

## Contribuições do estudo

**Principais resultados:**

Observou-se tendência de aumento de mortalidade por doença de Parkinson entre pessoas idosas de ambos os sexos, de todas as faixas etárias e em todas as regiões brasileiras no período.

**Implicações para os serviços:**

O aumento da mortalidade por doença de Parkinson implica maior demanda nos serviços de assistência à saúde e, consequentemente, contribui com o ônus financeiro relacionado ao diagnóstico e tratamento das pessoas com a doença.

**Perspectivas:**

Investir em pesquisas e tecnologias para diagnóstico oportuno, tratamento e recuperação da saúde são cruciais. Além disso, é necessário fomentar políticas de saúde que garantam acesso equitativo aos serviços especializados.

## INTRODUÇÃO

A doença de Parkinson é considerada a segunda doença degenerativa mais comum do sistema nervoso central.^1^ Seus principais sintomas são motores, incluindo bradicinesia, rigidez e tremor, geralmente começando a se manifestar entre 65 e 70 anos. No entanto, formas genéticas mais raras podem surgir antes dos 40 anos de idade.^1^

A prevalência de doença de Parkinson está em constante aumento, especialmente em países de baixa e média renda, possivelmente impulsionada pelo envelhecimento da população e pelo prolongamento da duração da doença.^2^ Além disso, estima-se que as taxas de incidência da doença de Parkinson variem entre 8 e 18 por 100 mil pessoas-ano.^3^ No entanto, dados sugerem que, dentro dos países, existem variações étnicas e regionais no risco da doença, possivelmente influenciadas por desigualdades no acesso aos cuidados de saúde.^4^

Um estudo, utilizando dados de mortalidade da Organização Mundial da Saúde, revelou que, no período de 1994 a 2019, as taxas de mortalidade por doença de Parkinson aumentaram em ambos os sexos globalmente; porém, o aumento foi mais acentuado no sexo masculino. De forma geral, a taxa de mortalidade por 100 mil habitantes subiu de 1,76, em 1994, para 5,67 em 2019, representando um aumento percentual de 222% ao longo de 25 anos.^5^

No Brasil, poucos estudos abordaram os indicadores de mortalidade relacionados à doença de Parkinson.^6,7^ Uma pesquisa conduzida no país, de 2010 a 2019, identificou 11.776 óbitos nesse período, com maior incidência no sexo masculino. No entanto, é importante observar que o estudo se limitou às capitais brasileiras e incluiu indivíduos com 40 anos ou mais.^6^ Em outro estudo, foi analisada a morbimortalidade da doença no Brasil de 2008 a 2020, considerando adultos e pessoas idosas. Os resultados revelaram uma média de 875 internações por ano, com uma maior taxa de mortalidade nos estados do Rio Grande do Sul e do Rio de Janeiro. Esses dados indicam um predomínio em pessoas idosas e no sexo masculino, sendo a região Sul do país a que apresenta a maior taxa de mortalidade.^7^

A análise da taxa de mortalidade em pessoas idosas ao longo de duas décadas, com técnicas estatísticas robustas, possibilitará identificar padrões em todo o Brasil. Esta investigação atualizada é crucial para destacar mudanças na ocorrência de óbitos relacionados à doença de Parkinson em pessoas idosas, sendo essencial para o planejamento de serviços de saúde. O estudo contribuirá para enriquecer o conhecimento existente, fornecendo informações valiosas para profissionais da saúde, pesquisadores e formuladores de políticas no contexto brasileiro.

Nesse contexto, o objetivo deste estudo foi descrever o perfil epidemiológico e analisar a tendência temporal das mortes por doença de Parkinson entre pessoas idosas no Brasil, de 2002 a 2021.

## MÉTODOS

Trata-se de um estudo descritivo sobre o perfil epidemiológico dos óbitos, e ecológico, analisando a tendência temporal das taxas de mortalidade por doença de Parkinson, segundo sexo, faixa etária e macrorregiões brasileiras, no período de 2002 a 2021, com dados do Sistema de Informação sobre Mortalidade (SIM), disponibilizados pelo Departamento de Informática do Sistema Único de Saúde. 

A análise foi conduzida no Brasil, onde, de acordo com o Instituto Brasileiro de Geografia e Estatística (IBGE), a área territorial é de 8.510.417,771 km², e a população totaliza aproximadamente 203.080.756 habitantes. Desse total, 32.113.490 são idosos com 60 anos ou mais de idade.

Foram incluídos os registros de óbitos em que a doença de Parkinson foi classificada como a causa básica, com o código G20 da Classificação Estatística Internacional de Doenças e Problemas Relacionados à Saúde (CID-10), para pessoas com 60 anos ou mais de idade. Dados e estimativas populacionais do Brasil e macrorregiões foram extraídos do Censo populacional de 2010, fornecidos pelo IBGE. Os dados foram extraídos em 14 de agosto de 2023.

Foram analisadas as seguintes variáveis:

- Sexo (masculino; feminino);
- Faixa etária (em anos: 60 a 69; 70 a 79; 80 ou mais);
- Raça/cor da pele (branca; preta; amarela; parda; indígena; ignorado);
- Escolaridade (nenhuma; de 1 a 3 anos; de 4 a 7 anos; de 8 a 11 anos; 12 anos ou mais; ignorada);
- Estado civil (solteiro; casado; viúvo; separado judicialmente; outro; ignorada);
- Macrorregião de residência (Norte; Nordeste; Sudeste; Sul; Centro-oeste);
- Ano de notificação (2002 a 2021);
- Taxa de mortalidade por doença de Parkinson.

As taxas de mortalidade foram obtidas dividindo-se o número de óbitos por doença de Parkinson em pessoas com 60 anos ou mais pelo número estimado de habitantes nessa faixa etária, conforme dados do IBGE para o mesmo período. Os resultados foram multiplicados por 100 mil habitantes. Foram também calculados os coeficientes de mortalidade geral na população idosa, segmentados por sexo, faixa etária e macrorregiões brasileiras.

Na análise descritiva do perfil dos óbitos por doença de Parkinson, foram consideradas as frequências absolutas (n) e relativas (%) das variáveis. Também foram descritas as taxas de mortalidade por doença de Parkinson. 

A análise de série temporal foi realizada empregando-se o modelo de regressão de Prais-Winsten para corrigir o efeito de autocorrelação de primeira ordem, frequentemente observado em dados populacionais.^8^ A variável dependente utilizada na análise foi o logaritmo das taxas de mortalidade por doença de Parkinson, enquanto a variável independente foi representada pelos anos da série temporal (2002-2021). As fórmulas propostas por Antunes e Cardoso^8^ foram aplicadas para calcular a variação percentual anual (VPA) e os intervalos de confiança de 95% (IC_95_%). 

Com relação à interpretação, um p-valor ≥ 0,05 foi interpretado como estabilidade da série temporal, ou seja, ausência de mudança significativa na tendência, enquanto um p-valor <0,05 foi interpretado como uma mudança significativa (variação positiva ou negativa) na tendência de aumento ou declínio da série temporal.^8^

A variação percentual (VP) da taxa de mortalidade, entre os anos de 2002 e 2021, foi calculada aplicando-se a seguinte fórmula:


$$VP=\frac{\text{Taxa de mortalidade em 2021 − Taxa de mortalidade em 2002}}{\text{Taxa de mortalidade em 2002}}\times 100$$

Os dados foram organizados com uso do programa Microsoft Office Excel 2019® e, em seguida, exportados para análise no *software* estatístico Stata 14 (StataCorp, Texas, EUA). 

Tratando-se de dados de domínio público e anonimizados, o projeto do estudo não necessitou ser submetido à avaliação de um Comitê de Ética em Pesquisa, conforme resolução nº 510, de 7 de abril de 2016, do Conselho Nacional de Saúde.

## RESULTADOS

No período de 2002 a 2021, foram registrados 57.723 óbitos por doença de Parkinson em indivíduos com 60 anos ou mais no Brasil, observando-se maior proporção de óbitos do sexo masculino (54,7%), na faixa etária de 80 anos ou mais (57,7%), entre idosos de raça/cor da pele branca (75,0%), de casados (41,5%) e entre aqueles com 1 a 3 anos de estudo (24,4%). Mais da metade dos óbitos ocorreram na região Sudeste (52,3%), seguida pelas regiões Sul (20,2%) e Nordeste (18,1%) (Tabela 1).

**Tabela 1:** Descrição dos óbitos por doença de Parkinson segundo características sociodemográficas e macrorregiões brasileiras, em indivíduos com idade ≥ 60 anos, Brasil, 2002-2021 (n = 57.723)

Variáveis	n	%
**Sexo**		
Masculino	31.592	54,7
Feminino	26.131	45,3
**Faixa etária (anos)**		
60 a 69	5.519	9,6
70 a 79	18.891	32,7
≥ 80	33.313	57,7
**Raça/cor da pele**		
Branca	43.310	75,0
Preta	1.763	3,1
Amarela	517	0,9
Parda	9.778	16,9
Indígena	37	0,1
Ignorada	2.318	4,0
**Estado civil**		
Solteiro	5.990	10,4
Casado	23.944	41,5
Viúvo	21.705	37,6
Separado judicialmente	2.532	4,4
Outro	524	0,9
Ignorado	3.028	5,2
**Escolaridade (anos)**		
Nenhuma	7.399	12,8
De 1 a 3	14.092	24,4
De 4 a 7	10.725	18,6
De 8 a 11	7.454	12,9
≥ 12	5.323	9,2
Ignorada	12.730	22,1
**Macrorregião de residência**		
Norte	1.784	3,1
Nordeste	10.425	18,1
Sudeste	30.188	52,3
Sul	11.675	20,2
Centro-Oeste	3.651	6,3
Brasil	57.723	100,00

A Tabela 2 apresenta as taxas anuais de mortalidade por doença de Parkinson no período do estudo. Observou-se aumento nos coeficientes segundo todas as características analisadas. O coeficiente de mortalidade na população do sexo feminino aumentou de 6,6 por 100 mil habitantes, em 2002, para 12,0 por 100 mil habitantes, em 2021, enquanto na população do sexo masculino se elevou de 10,0 por 100,000, em 2002, para 19,6 por 100 mil habitantes, correspondendo a um aumento de 96,0% neste sexo (Tabela 2).

**Tabela 2:** Coeficiente de mortalidade por doença de Parkinson em indivíduos com idade ≥60 anos, de acordo com sexo, faixa etária e macrorregiões brasileiras, Brasil, 2002-2021

	2002	2003	2004	2005	2006	2007	2008	2009	2010	2011	2012	2013	2014	2015	2016	2017	2018	2019	2020	2021	2021/ 2002 (%)^a^
**Sexo**																					
Masculino	10,0	10,6	11,7	13,0	14,1	14,4	14,0	13,7	14,3	15,7	16,1	15,8	16,4	15,9	16,8	17,0	17,9	19,4	19,2	19,6	+96,0
Feminino	6,6	7,3	7,5	9,0	10,2	9,5	9,1	10,2	10,4	9,9	10,6	10,4	11,2	10,7	10,9	10,9	12,2	12,2	12,0	12,0	+81,8
**Faixa etária (anos)**																					
60 a 69	1,9	1,7	2,1	2,0	2,0	2,1	2,2	2,2	2,2	2,0	2,0	2,1	2,1	2,1	2,3	2,2	2,4	2,6	2,4	2,7	+42,1
70 a 79	9,5	10,6	11,1	12,7	13,6	13,4	12,7	12,4	13,2	14,3	14,6	13,4	14,9	12,9	14,0	14,3	15,1	15,8	15,8	15,8	+66,3
≥ 80	34,5	37,0	37,0	43,8	49,3	47,5	44,6	47,9	48,2	49,2	52,6	53,3	55,1	55,6	56,1	56,1	60,7	63,0	62,2	61,7	+78,8
**Macrorregião**																					
Norte	3,5	3,0	3,7	5,5	6,5	5,2	5,8	6,5	5,4	7,3	7,5	6,9	7,1	8,9	7,8	8,9	10,0	8,6	12,2	9,9	+182,9
Nordeste	3,4	3,9	4,5	6,2	6,8	7,5	8,4	7,3	8,2	8,7	8,8	9,2	9,8	10,3	10,8	10,7	10,5	11,9	12,8	12,8	+276,5
Sudeste	10,3	11,0	11,4	13,1	14,4	13,9	12,6	14,0	14,7	14,2	14,9	14,2	15,4	14,3	14,7	14,4	15,5	16,7	15,3	15,9	+54,4
Sul	11,1	12,0	12,8	13,8	15,1	14,9	14,0	14,2	14,2	15,6	16,2	16,0	16,0	14,4	16,4	16,9	19,7	18,1	18,6	19,4	+74,8
Centro-Oeste	7,8	10,0	10,7	10,4	11,9	10,6	10,7	11,7	10,3	11,5	13,1	13,5	13,0	14,3	12,9	14,4	15,8	17,6	16,5	15,1	+93,6
Brasil	8,1	8,8	9,3	10,8	11,9	11,7	11,3	11,7	12,1	12,5	13,0	12,8	13,5	13,0	13,5	13,6	14,7	15,4	15,2	15,4	+90,1

a) Aumento (+) ou diminuição (−) percentual, comparando-se os anos de 2021 e 2002.

Na faixa etária de 80 anos ou mais, os coeficientes de mortalidade passaram de 34,5 por 100 mil habitantes, em 2002, para 61,7 por 100 mil habitantes, em 2021, representando um incremento superior a 78 pontos (p.p.) no período. Em relação ao percentual de aumento das taxas de mortalidade entre o final e o início da série histórica, a região Nordeste, com um incremento de 276,5% dos coeficientes de mortalidade, se destacou (Tabela 2).

Ao analisar os coeficientes de mortalidade por doença de Parkinson nas regiões brasileiras, destacou-se a região Sul, que apresentou os maiores coeficientes de mortalidade em todo o período do estudo (exceto no ano de 2010), atingindo 19,4 mortes a cada 100 mil habitantes, em 2021, seguida pela região Sudeste, onde se registravam 10,3 mortes por 100 mil habitantes, em 2002, e 15,9 a cada 100 mil habitantes, em 2021 (Figura 1).

**Figura 1:** Série temporal dos coeficientes de mortalidade por doença de Parkinson em indivíduos com idade ≥ 60 anos, no Brasil e macrorregiões brasileiras, 2002-2021

A análise de tendência temporal mostrou tendência de aumento na mortalidade nos dois sexos, em todas as faixas etárias e em todas as regiões brasileiras. A maior VPA foi observada no sexo masculino (3,32%; IC_95_% 2,49;4,16), na faixa etária de 70 a 79 anos (4,93%; IC_95_% 2,98;6,91) e na região Nordeste (6,79%; IC_95_% 4,35;9,30), seguida pela região Norte, com uma variação de 5,85% (IC_95_% 4,45;7,28) (Tabela 3).

**Tabela 3:** Série temporal dos coeficientes, com variação percentual anual (%) e intervalos de confiança de 95% (IC95%), de mortalidade por doença de Parkinson em indivíduos com idade ≥ 60 anos, de acordo com sexo, faixa etária e macrorregiões brasileiras, Brasil, 2002-2021

Variável	Coeficiente (logaritmo)	Erro-padrão	% (IC_95_ **%**)	p-valor	Interpretação
**Sexo**					
Masculino	0,01	0,00	3,32 (2,49;4,16)	<0,001	Aumento
Feminino	0,01	0,00	2,81 (1,80;3,82)	<0,001	Aumento
**Faixa etária (anos)**					
60 a 69	0,00	0,00	1,49 (0,85;2,15)	<0,001	Aumento
70 a 79	0,02	0,00	4,93 (2,98;6,91)	<0,001	Aumento
≥ 80	0,01	0,00	2,93 (2,21;3,65)	<0,001	Aumento
**Macrorregião**					
Norte	0,02	0,00	5,85 (4,45;7,28)	<0,001	Aumento
Nordeste	0,03	0,00	6,79 (4,35;9,30)	<0,001	Aumento
Sudeste	0,00	0,00	1,87 (1,07;2,68)	<0,001	Aumento
Sul	0,01	0,00	2,46 (1,77;3,16)	<0,001	Aumento
Centro-Oeste	0,01	0,00	3,15 (2,44;3,87)	<0,001	Aumento
Brasil	0,01	0,00	3,12 (2,18;4,07)	<0,001	Aumento

## DISCUSSÃO

No presente estudo, observou-se predomínio dos óbitos registrados como causa da doença de Parkinson do sexo masculino, nas faixas etárias mais elevadas e com baixa escolaridade. Os coeficientes de mortalidade mais elevados foram registrados nas regiões Sul e Sudeste, bem como na faixa etária de 80 anos ou mais. Os resultados da análise de tendência temporal indicaram aumento da mortalidade pela doença em ambos os sexos, em todas as faixas etárias e em todas as macrorregiões brasileiras, com destaque para a maior variação percentual anual no sexo masculino, na faixa etária de 70 a 79 anos, e nas regiões Nordeste e Norte.

Maior proporção de óbitos e coeficientes de mortalidade mais elevados foram observados no sexo masculino em comparação com o feminino. Estes resultados corroboram dados de estudos anteriores que analisaram óbitos por doença de Parkinson em diversos períodos, tanto no âmbito nacional,^6,7^ quanto globalmente.^5^ Essa diferença pode ser explicada por vários fatores. Mulheres produzem naturalmente estrogênios, hormônios que demonstraram ter um efeito protetor contra o desenvolvimento da doença. Além disso, homens geralmente apresentam os primeiros sintomas da doença em uma idade mais jovem do que as mulheres, o que significa que têm um período mais longo para que a doença progrida, resultando em um aumento na taxa de mortalidade.^5^

Neste estudo, foi observado aumento de mais de 78 p.p. nos coeficientes de mortalidade pela doença na faixa etária de 80 anos ou mais, no período analisado. Este achado está em consonância com outros estudos brasileiros que utilizaram as mesmas fontes de dados em diferentes períodos, bem como com as tendências internacionais.^5,6,7^ De fato, observa-se uma tendência de aumento na prevalência da doença com a idade, resultando em sintomas mais severos e maior risco de mortalidade associado à doença.^9,10^ Este fenômeno é influenciado pelo aumento da expectativa de vida e pelo envelhecimento da população.^5,11^ Além disso, comorbidades como demência, fragilidade e quedas, comuns nessa faixa etária, são importantes preditores de mortalidade em pessoas idosas com a doença de Parkinson.^12^

Observou-se relação inversamente proporcional entre a mortalidade por doença de Parkinson e o grau de escolaridade, tendo 24,4% dos óbitos ocorrido em pessoas com até três anos de estudo. Isso contrasta com outros trabalhos, que evidenciaram que o nível educacional e QI elevados são fatores de risco para a doença de Parkinson.^13,14^ Ademais, nossos resultados podem ser parcialmente atribuídos ao elevado número de óbitos nos quais o nível de escolaridade não foi informado. Destaca-se que aproximadamente 22,1% dos registros não apresentavam informações sobre o nível educacional. A falta de preenchimento adequado dessas informações no SIM pode ter influenciado o perfil identificado.

A região Sul se destacou do país por registrar consistentemente os maiores coeficientes de mortalidade por doença de Parkinson no período estudado, seguida pela região Sudeste. Estes resultados concordam com dados de um estudo que analisou as taxas de mortalidade pela doença em população de indivíduos com 30 anos ou mais de idade, no Brasil, no período de 2008 a 2020, que encontrou maiores taxas de mortalidade por doença de Parkinson nessa região.^7^ Um estudo realizado somente nas capitais brasileiras identificou uma taxa de mortalidade mais elevada na região Sudeste durante o período de 2010 a 2019, em indivíduos com 40 anos ou mais.^6^

O Brasil, devido à sua extensão territorial e diversidade regional, apresenta notáveis discrepâncias no desenvolvimento socioeconômico, demografia, acesso a serviços de saúde (melhor diagnóstico) e expectativa de vida.^15^ Em destaque, as regiões Sul e Sudeste são caracterizadas por um elevado Índice de Desenvolvimento Humano, que considera fatores como renda e longevidade.^16^ Essas disparidades inter-regionais podem influenciar os padrões de envelhecimento, que se refletem nas diferenças regionais de mortalidade por Parkinson identificadas neste estudo. 

Observou-se tendência de aumento de mortalidade pela doença em ambos os sexos, em todas as faixas etárias e em todas as regiões brasileiras. Esse resultado pode ser parcialmente explicado pela melhoria da qualidade dos registros. Isso pode influenciar as declarações de óbito, contribuindo para o aumento contínuo da mortalidade por doença de Parkinson.^17^ No entanto, torna-se importante destacar outras possíveis explicações. Globalmente, a doença de Parkinson apresentou a maior taxa de crescimento entre as condições neurológicas, e a crescente prevalência pode estar causando um impacto significativo na mortalidade. Esse aumento é principalmente atribuído ao envelhecimento da população, a avanços significativos nos biomarcadores de diagnóstico e tratamentos modificadores da doença e a causas ambientais.^5^

Ao analisarmos os índices de VPA de óbitos por doença de Parkinson encontrados no estudo, observamos que a região Nordeste apresentou a maior VPA, atingindo 6,79%, seguida pela região Norte, com uma variação de 5,85%. Durante o período de 2000 a 2014, as regiões Norte e Nordeste destacaram-se pela abertura de novas fronteiras agrícolas e experimentaram os maiores incrementos nas vendas de agrotóxicos, tendo a região Norte apresentando expressivo aumento de 99,78%, seguida de perto pela região Nordeste, que registrou um incremento significativo de 97,50%.^27^ O grau de intensidade de uso nos estados das regiões Norte e Nordeste continuou aumentando até 2017.^20^ Além disso, outras possíveis explicações para a maior VPA nestas regiões foram mencionadas anteriormente, como maior acesso aos serviços de saúde e capacidade diagnóstica,^15^ melhor registro nos sistemas de informação e envelhecimento populacional.

Em relação ao sexo, houve incremento na VPA de óbitos por doença de Parkinson tanto na população masculina (3,32%) quanto feminina (2,81%). Embora a doença seja mais prevalente na população masculina em relação à feminina, conforme evidenciado neste trabalho, como também em estudo no âmbito global, ressalta-se que foi igualmente observado aumento significativo na VPA nesta última. Evidências demonstram a existência de diferenças entre os sexos no fenótipo clínico, biomarcadores e manejo terapêutico da doença de Parkinson.^28,29^ Um estudo de revisão constatou que essa doença em mulheres começa com um fenótipo mais benigno, provavelmente devido ao efeito dos estrogênios. No entanto, à medida que a doença progride, as mulheres têm um risco maior de desenvolver complicações altamente incapacitantes relacionadas ao tratamento, bem como discinesia, em comparação com os homens.^29^ Além disso, as mulheres têm menor chance de receber tratamento eficaz,^28^ maior probabilidade de gravidade da doença e mais comorbidades do que os homens.^30^ Esses fatores em conjunto podem contribuir para a explicação do aumento na VPA identificado também no público feminino.

As informações de mortalidade desempenham um papel fundamental no planejamento e na distribuição de recursos em saúde pública. Esses dados são essenciais para subsidiar a elaboração de políticas públicas voltadas para doenças neurodegenerativas, permitindo a adaptação de estratégias de assistência à saúde de acordo com as tendências identificadas. Na presente pesquisa, observou-se um aumento nos coeficientes de mortalidade pela doença em todas as características analisadas. Diante desse cenário, torna-se crucial investir em estratégias que permitam o diagnóstico precoce, tratamento efetivo e apoio integral aos portadores da doença. 

Quanto às limitações deste estudo, é importante destacar que o uso de dados secundários pode apresentar desafios, devido a possíveis erros e omissões no registro das informações e/ou subnotificação da doença. Uma falha significativa no preenchimento da variável escolaridade foi identificada, abrangendo 22,1% dos registros. Isso pode ter ocasionado um viés de classificação, contribuindo para o maior percentual de óbitos detectados entre indivíduos com menor escolaridade. Ainda, destaca-se a impossibilidade de realizar análises de tendência para outras características, como raça/cor da pele e estado civil, devido à ausência de estimativas populacionais detalhadas que serviriam como denominadores necessários para o cálculo das taxas de mortalidade. No entanto, a pesquisa se destaca pela utilização de métodos robustos de análise da tendência temporal das taxas de mortalidade e por compreender extenso período de observação, de 2002 a 2021. Além disso, as análises foram estratificadas por sexo, faixa etária e macrorregiões brasileiras, permitindo a análise mais detalhada dos padrões de mortalidade nestes subgrupos da população.

Conclui-se que os óbitos por doença de Parkinson predominaram no sexo masculino, em faixas etárias mais elevadas, e na população de raça/cor da pele branca, com tendência de aumento da mortalidade em ambos os sexos, todas as faixas etárias e macrorregiões. Assim, são necessários investimentos em pesquisas e tecnologias para diagnóstico precoce, tratamento eficaz e recuperação da saúde. Além disso, é crucial implementar políticas de saúde que garantam o acesso equitativo aos serviços especializados, visando oferecer assistência de qualidade a todos os pacientes afetados pela doença de Parkinson.
